# Adventitial cystic disease of the radial artery

**DOI:** 10.1590/1677-5449.012617

**Published:** 2018

**Authors:** Adriano Carvalho Guimarães, Ricardo Herkenhoff Moreira, Walter Junior Boim de Araujo

**Affiliations:** 1 V&P Health Excelência Médica, Santo Antônio da Platina, PR, Brasil.; 2 Hospital Nossa Senhora da Saúde, Santo Antônio da Platina, PR, Brasil.; 3 Instituto da Circulação, Curitiba, PR, Brasil.

**Keywords:** adventitial cystic disease, radial artery, cysts, doença cística adventicial, artéria radial, cistos

## Abstract

Adventitial cystic disease (ACD) of the radial artery is a rare condition, with few cases described in the literature. We report the case of a 62-year-old white male with a history of diabetes, hypertension, and chronic kidney disease with indications for renal replacement therapy who was found to have a cystic lesion of the radial artery while undergoing surgical creation of an arteriovenous fistula. The surgical technique adopted was resection of the cystic segment and preservation of the radial artery. Fistula creation was completed successfully. Early diagnosis and appropriate treatment of ACD are effective, and can prevent complications and recurrence.

## Resumo

A doença cística adventicial (DCA) da artéria radial é uma condição rara, com poucos casos descritos na literatura. Relatamos o caso de um paciente do sexo masculino, 62 anos, branco, diabético, hipertenso, com insuficiência renal crônica e indicação para terapia substitutiva renal, em quem foi encontrada uma lesão cística da artéria radial durante operação para confecção de fistula arteriovenosa para hemodiálise. Após a dissecção da artéria radial, ficou evidenciado um importante envolvimento do vaso por uma formação cística. A técnica cirúrgica adotada foi a ressecção do segmento cístico comprometido e preservação da artéria radial. A confecção da fistula arteriovenosa foi realizada com sucesso. O diagnóstico precoce e o tratamento adequado da DCA mostram se eficientes e podem prevenir complicações e recidivas.

## INTRODUCTION

 Adventitial cystic disease (ACD) is a rare, non-atherosclerotic arteriopathy, predominantly involving vessels of the lower extremity, of which the popliteal artery is most commonly affected. 1

 It is more common in men, with a 5:1 male-to-female ratio. Since ACD was first described in 1947, our understanding of this disease has largely been based on individual case reports and literature reviews. The overall prevalence of this arteriopathy remains unknown; an estimated 500 cases have been reported. 2 Its incidence in the radial artery is even rarer, with only a few cases described in the literature. 3

 ACD is characterized by a buildup of mucinous or gelatinous matter in the adventitial layer of the vessel wall. Its etiology remains controversial. Trauma, associated systemic processes, or development of mucin-secreting cells within the adventitia have all been proposed as hypotheses; more recently, evidence has arisen in support of the synovial theory. 4

 Patients with ACD of the radial artery may present with a pulsatile mass, though symptoms of luminal narrowing or occlusion may emerge due to compression of the involved artery. Pain, weakness, paresthesias, and even ischemia of the hand have been reported. 3 ^,^ 5 ^,^ 6

 Several different treatment options have been described, such as resection of the compromised cystic segment, percutaneous cyst aspiration, resection and reconstruction of the affected artery segment, and endovascular treatment. 

 Written informed consent was obtained from the patient for publication of this case report and the accompanying images. 

## CASE DESCRIPTION

 A 62-year-old white male with a history of diabetes, hypertension, and chronic kidney disease with indications for renal replacement therapy presented for surgical creation of an arteriovenous fistula for hemodialysis. After assessment by the Vascular Surgery team, a radiocephalic arteriovenous fistula in the left upper extremity was deemed most appropriate. 

**Figure 1:**
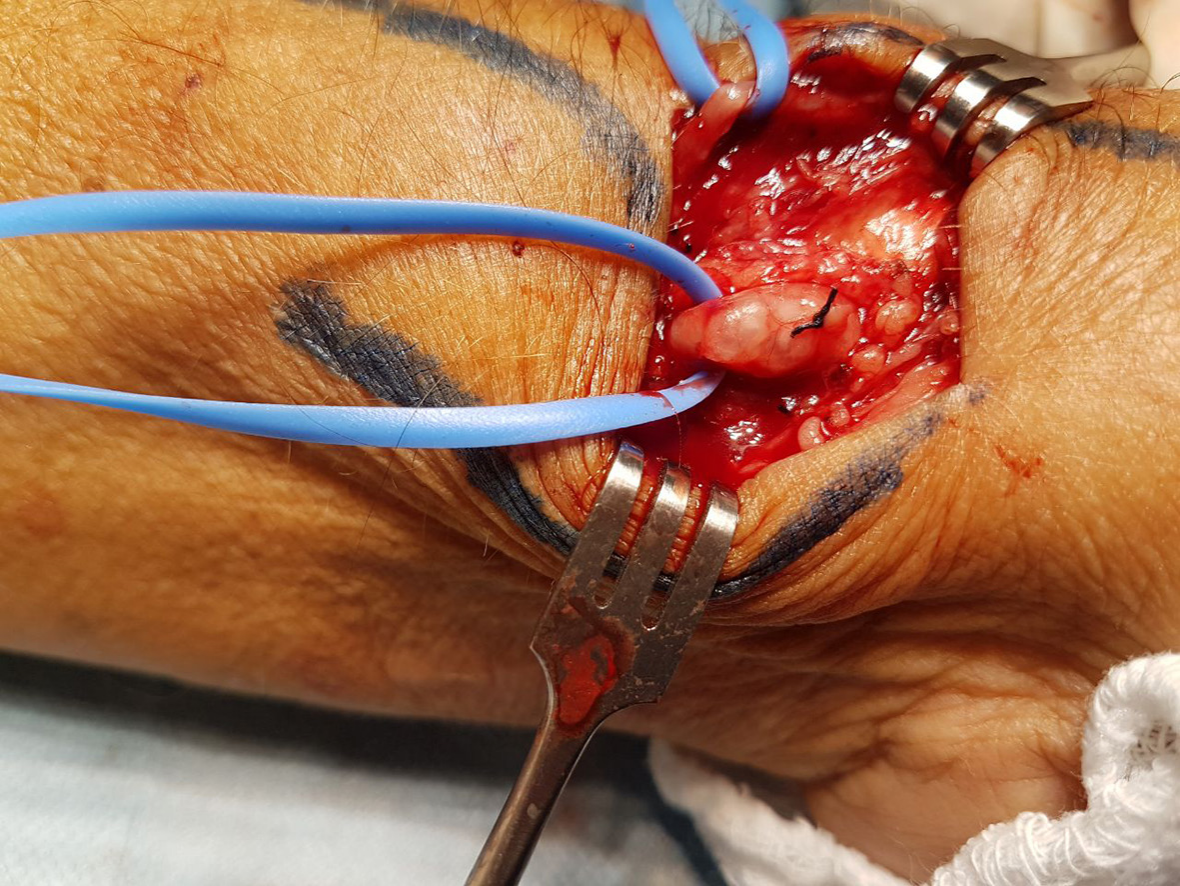
Cystic lesion involving the radial artery, with no clear boundaries between the cysts and the underlying vessel.

**Figure 2:**
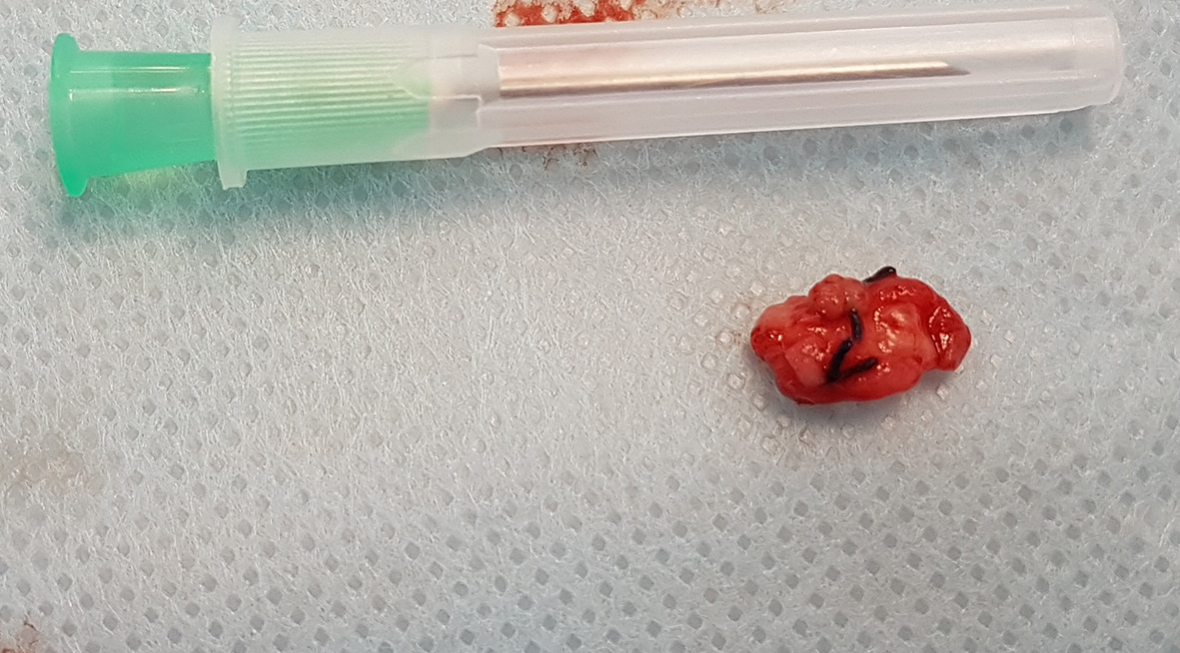
Resected surgical specimen of the cystic segment.

**Figure 3:**
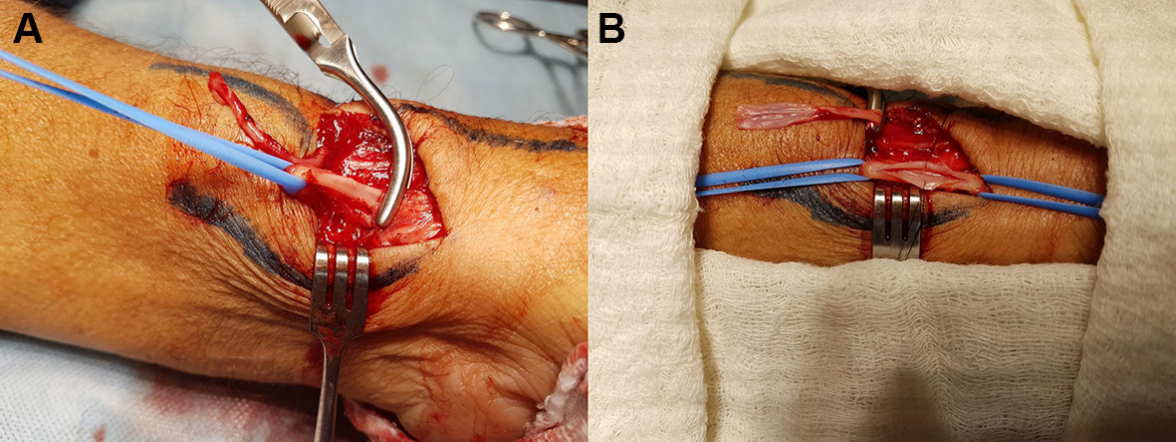
(A) Appearance of the radial artery after cyst resection; (B) Radial arteriotomy and preparation of the cephalic vein for creation of radiocephalic arteriovenous fistula.

 During surgical creation of the fistula in the left wrist, dissection of the radial artery revealed a large cystic formation enmeshed in the vessel, with no clear borders between the cyst and artery ( Figure 1 ), but with a strong local pulse. Slight adhesions between the vessel and cyst allowed the latter to be dissected free of the artery without rupture or major leakage of cyst content. The surgical technique adopted was resection of the cystic segment ( Figure 2 ) and preservation of the radial artery, enabling successful fistula creation ( Figure 3 ). 

**Figure 4:**
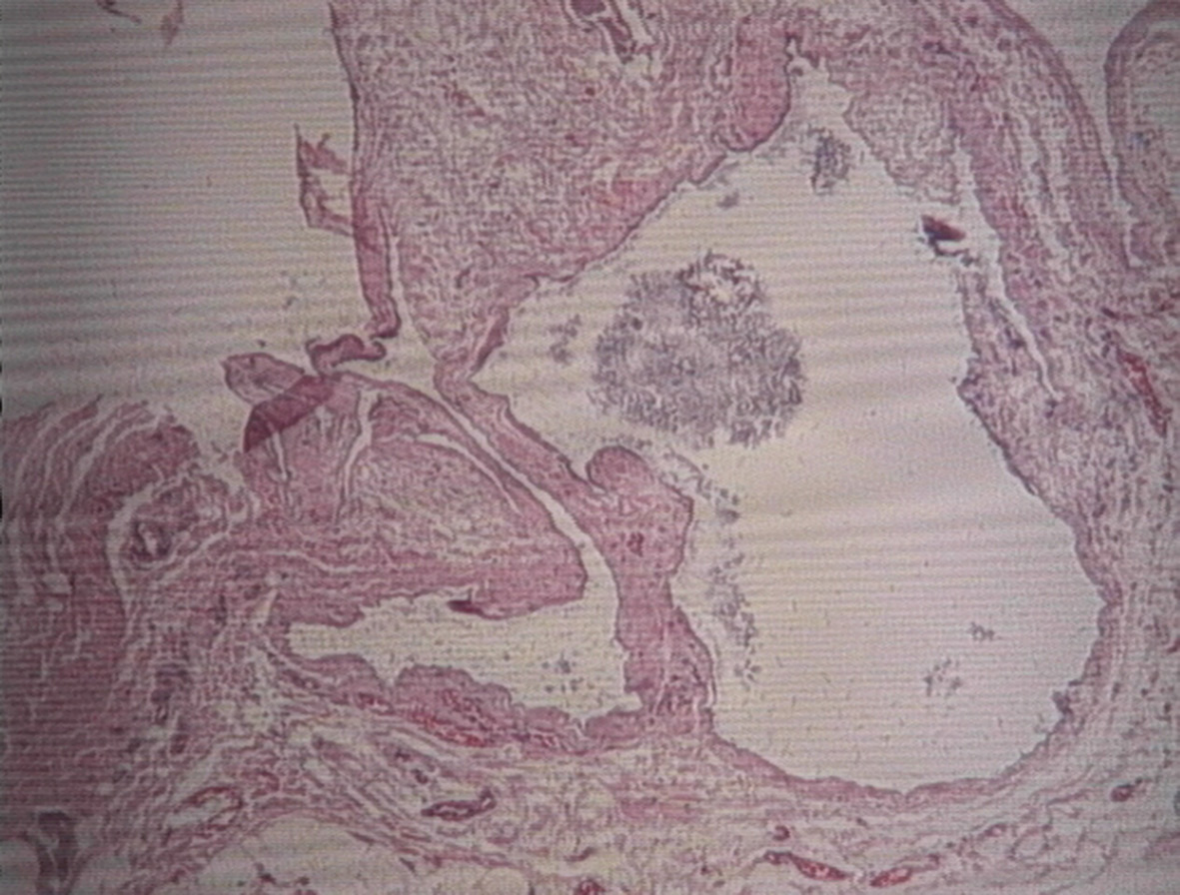
Histopathological analysis of a hematoxylin and eosin (H&E)-stained section, confirming the presence of a cystic lesion of the arterial wall. Original magnification ×20.

**Figure 5:**
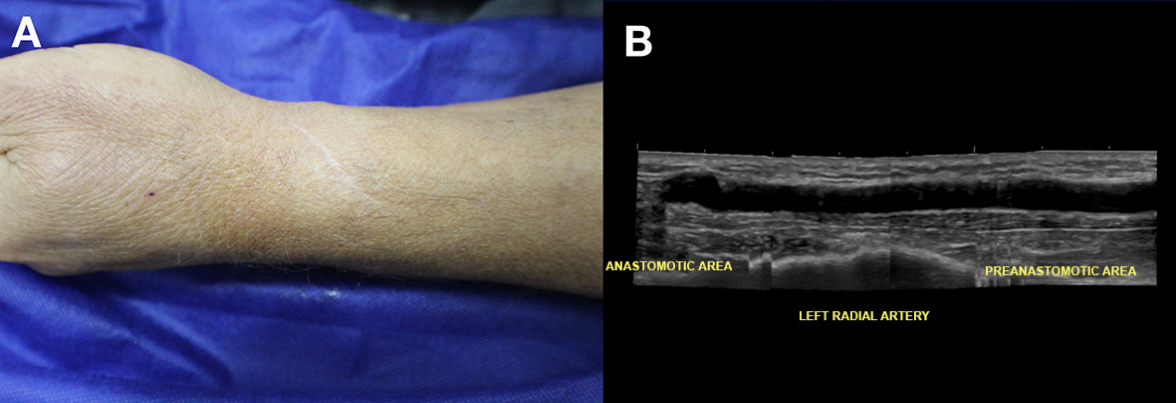
(A) Healing of surgical wound and absence of edema in the left upper extremity 1 year after creation of an arteriovenous fistula; (B) B-mode extended panoramic image showing no degenerative changes in the radial artery.

 Gross pathological examination of the surgical specimen revealed a viscous substance within the cystic mass. Histopathological analysis confirmed the presence of a cystic lesion of the arterial wall ( Figure 4 ). The patient is being followed by a multidisciplinary team. The fistula is currently functional, and follow-up echo-Doppler performed 1 year after the procedure revealed no signs of artery degeneration ( Figure 5 ). 

## DISCUSSION

 The first case of ACD was reported in 1947, involving the external iliac artery. 7 However, the popliteal artery is most commonly affected; the first such case was published in 1954. 8 The femoral, 9 axillary 10 and radial arteries may also be affected, although the radial artery is a particularly rare site, accounting for only 2.5% of ACD cases. 2 Venous ACD has also been reported. 11

 The etiology of ACD is highly controversial and remains open to debate. Proposed theories include: (A) Repetitive trauma leading to cystic degeneration. This theory is contested because most patients with the condition have no history of trauma; 1 ^,^ 2 ^,^ 12 (B) Manifestation of a systemic condition. This theory is inconsistent, since no association has been demonstrated between ACD and any other organs or systems; (C) Development of mesenchymal cells within the adventitia. This theory is essentially unjustifiable, since recurrence has been observed even after complete excision of the cyst; and (D) The result of direct communication with an adjacent space. The discovery that ACD cysts are pedunculated and connected to the adjacent joint and synovial-like histological findings of this condition provide support for this theory. The gelatinous substance found within the cysts is grossly identical to the content of normal joint capsules. 4 ^,^ 13 ^-^ 15

 Torres-Blanco et al. reported a case of radial artery ACD in which a pedicle was found passing through the superficial palmar branch of the radial artery and connecting to the wrist joint, thus providing additional evidence in favor of the synovial hypothesis. 16

 As ACD is considered a rare disease entity, there are no guidelines for its management. Several options have been proposed, including nonsurgical management, cyst aspiration (percutaneous or open), angioplasty, or surgical treatment. Surgical options include incision and decompression, isolated cyst resection, venous or synthetic patch repair, or venous or synthetic graft reconstruction. 17 ^,^ 18

 Motaganahalli et al. retrospectively analyzed 10 years of information from a standardized database covering 14 facilities, in the largest-ever published series of ACD cases. Of the 47 patients assessed, only three (6.38%) had ACD of the radial artery. The authors concluded that, among the currently available treatment options, cyst resection and graft reconstruction is associated with the highest likelihood of symptom resolution and a low rate of reintervention due to cyst recurrence. 19

 In the case described herein, ACD of the radial artery was diagnosed during surgical creation of an AV fistula for hemodialysis. As the artery had to be preserved for the fistula, the decision was made to simply resect the cystic segment, thus allowing completion of the surgical procedure originally planned. 

 Adventitial cystic disease of the radial artery is quite an unusual entity. Detection of a pulsatile mass in the anatomical course of this artery should raise clinical suspicion of ACD. Although its etiology remains controversial, early diagnosis and appropriate treatment of ACD are effective, and can prevent complications and recurrence. 
